# Supplementary material for: Adjusting dialysis dose (Kt) scaled to Body Surface Area (BSA) could be a more logical approach
Source: PLoS One. 2024 Oct 7;19(10):e0311175. doi: 10.1371/journal.pone.0311175 (PMC11458025; doi:10.1371/journal.pone.0311175)
Supplement: Attachment — Submitted filename: Response to reviewers R1.docx [file pone.0311175.s002.docx]

**Response to reviewers**

Dear editor and reviewers of PLoS One:

Our reference: PONE-D-24-24065

Title: Adjusting dialysis dose (Kt) scaled to Body Surface Area (BSA) could be a more logical approach

By: Liu Wei et al

Thank you very much for your letter and for the editors’ and reviewers’ comments concerning our manuscript entitled “Adjusting dialysis dose (Kt) scaled to Body Surface Area (BSA) could be a more logical approach” (ID: PONE-D-24-24065). These comments are of great reference value to the revision and improvement of our paper and have important guiding significance to our researches. The main corrections in the paper and the responds to the reviewer’s comments are as flowing:

Reviewer #1: The manuscript presents an interesting approach to scaling dialysis dose (Kt) to body surface area (BSA) instead of the conventional urea distribution volume (V) for a more accurate reflection of metabolic activity. The study is well-structured, with a clear abstract and comprehensive results.

The manuscript states that linear regression analysis was applied, but it does not provide details about the regression model assumptions, the goodness-of-fit metrics, or the potential multicollinearity issues. Including information about the model diagnostics (residual analysis, R-squared values) and any steps taken to validate the regression model would strengthen the analysis.

Author's reply：The article adds the corresponding content as follows:

“Femle，Kt/V and Kt/BSA regression equation：Kt/BSA_(f)_=0.34+0.73×Kt/V_(f)_(Analysis of variance on the model result:F=107.59,P<0.01; r=0.77,R^2^=0.60,Std. Error of the Estimate is 0.11,Durbin-Watson Statistic is 2.15);Male，Kt/V and Kt/BSA regression equation：Kt/BSA_(m)_=0.30+0.87×Kt/V_(m)_(Analysis of variance on the model result:F=384.70,P<0.01; r=0.86,R^2^=0.74,Std. Error of the Estimate is 0.14,Durbin-Watson Statistic is 1.93)”

and

”Femle，BSA and V regression equation：V_(f)_=-5.01+21.92×BSA_(f)_(Analysis of variance on the model result:F=90.58 ,P<0.01 ; r=0.74,R^2^=0.55,Std. Error of the Estimate is 2.24 ,Durbin-Watson Statistic is 2.19) ;Male，BSA and V regression equation：V_(m)_=-22.29+34.91×BSA_(m)_ (Analysis of variance on the model result:F=418.19 ,P<0.01 ; r=0.87,R^2^=0.76,Std. Error of the Estimate is 3.29,Durbin-Watson Statistic is 1.95).”

Reviewer #2: In this single-centre cross-sectional study, the researchers investigated the efficacy of hemodialysis, as measured by Kt/V versus Kt/BSA, in 211 patients with end-stage chronic kidney disease undergoing chronic hemodialysis. The focus of the two dialysis parameters for adequacy was on gender comparison. The inclusion and exclusion criteria are clear. The study was approved by the ethics committee and all participants signed a written informed consent. Most of the participants had an arteriovenous fistula as vascular access. The researchers found significantly higher Kt/V values in women than in men and after normalisation from Kt to BSA, the difference between the two sexes was no longer statistically significant.

The manuscript is well written, the entire text needs to be reviewed by a native English speaker.

I have the following comments, questions, suggestions:

1. When were the blood samples for the Kt/V measurements taken? At the first, second or third hemodialysis session of the week?

Author's reply：In the article, Kt/V represents the dose for a single treatment,The blood samples were taken randomly.The measurement time is not fixed.

2. Do all patients have the same weekly schedule for dialysis sessions?

Author's reply：Yes,the hemodialysis regimen mandates a thrice-weekly session schedule, with each session lasting four hours.

The article added "Hemodialysis Treatment Protocol" related content as follows:

3. “3.Hemodialysis Treatment Protocol: Hemodialysis is performed a thrice-weekly session schedule, with each session lasting four hours. Blood flow rate is set between 200-300 mL/min, and dialysate flow is 500 mL/min. A standardized, glucose-free, bicarbonate-based dialysate is used for hemodialysis procedures. Anticoagulation with calcium-based heparin prevents clots, The use of single-use dialyzers and dialysis tubing is mandatory to uphold the highest standards of patient safety.”
3. Data on the duration of the hemodialysis session of the participants at the time of blood sampling is missing.

Author's reply：The article adds the corresponding content as follows:

4. Data on the type of dialysis (hemodialysis, hemofiltration, hemodiafiltration) are missing.

Author's reply：We only have two types of dialysis(hemodialysis(HD) and hemodiafiltration(HDF)).The article adds the corresponding content as follows:

5. Minor comments:

Table 1: SBA is incorrect, it is BSA

Relationship between Kt/V and Kt/BSA by gender (not by sex)

Author's reply：The corresponding part of the article has been revised.

Once again, thank you very much for your comments and suggestions.

2024-08-30
